# Understanding abortion legality and trimester of abortion care in Ohio, West Virginia and Kentucky, three abortion‐restrictive states

**DOI:** 10.1111/psrh.12284

**Published:** 2024-09-17

**Authors:** Annamarie L. Beckmeyer, Jeremy A. Brenner‐Levoy, B. Jessie Hill, Tamika C. Odum, Abigail Norris Turner, Alison H. Norris, Danielle Bessett, Katherine L. Rivlin

**Affiliations:** ^1^ Department of Obstetrics and Gynecology Ohio State University College of Medicine Columbus Ohio USA; ^2^ Department of Sociology University of Cincinnati Cincinnati Ohio USA; ^3^ School of Law Case Western Reserve University Cleveland Ohio USA; ^4^ Behavioral Science Department, Blue Ash College University of Cincinnati Cincinnati Ohio USA; ^5^ Division of Epidemiology, College of Public Health Ohio State University Columbus Ohio USA; ^6^ Department of Obstetrics and Gynecology University of Chicago Chicago Illinois USA

**Keywords:** abortion, law/legal issues

## Abstract

**Introduction:**

In the era of Dobbs, legality of abortion care in the United States depends upon state law. Even before Dobbs, while abortion remained legal mounting restrictions and debate surrounding legal abortion could have led to confusion about abortion legality and discouraged patients from accessing legal abortion. We hypothesized an association between believing abortion is illegal or uncertainty about legality with later timing of abortion care.

**Methods:**

We surveyed patients seeking abortion care in Ohio, West Virginia, and Kentucky from April 2020 to April 2021. We asked about their understanding of abortion legality at the time they were first deciding to have an abortion. Using unconditional logistic regression models, we examined associations between beliefs about abortion legality (measured as belief that abortion is legal or sometimes legal versus. illegal or unsure) and timing of abortion care (measured as trimester of abortion).

**Results:**

Over half (57%) of the 1,479 patients who met eligibility criteria and completed the survey believed abortion was always legal, 21% thought abortion was sometimes legal, 12% believed abortion was illegal, and 10% did not know. Most (92%) had a first trimester abortion (<14 weeks gestation). Belief that abortion was illegal, or uncertainty about abortion legality, was not significantly associated with second trimester abortion care (unadjusted odds ratio [uOR]: 0.78, 95% confidence interval [CI]: 0.50–1.20). This association did not change meaningfully after adjusting for demographic and clinical variables (adjusted OR [aOR]: 0.83, 95% CI: 0.51–1.33).

**Discussion:**

More than one in five patients presenting for abortion care in three abortion‐restrictive states prior to Dobbs erroneously believed that abortion was illegal or were unsure. Understanding of legality was not significantly associated with timing of abortion care. These misunderstandings could escalate under Dobbs.

## INTRODUCTION

With the 2022 Supreme Court decision in Dobbs v. Jackson Women's Health Organization (Dobbs)^1^ and the loss of federal protection of abortion access, the legality of abortion in the United States now depends on state law. Many states have heavily restricted or banned abortion care.^2^ Even in the decade before Dobbs, states enacted an unprecedented number of abortion regulations—with a record of 106 in 2021 alone.^3^ Common restrictions included mandating waiting periods, banning specific abortion procedures, and setting gestational age limits on abortion.^4^

The patchwork of pre‐ and post‐Dobbs restrictions and debate surrounding what constitutes a legal abortion can lead to patient confusion and chill access to legal abortion care.^5^, ^6^, ^7^ Ambiguous abortion regulations may intentionally generate misunderstanding, increase abortion stigma, and influence whether, how, and when patients seek care.^7^, ^8^ In a pre‐Dobbs survey by Kavanaugh and colleagues, participants who answered incorrectly about the legality of abortion were also more likely to associate abortion with false risks, such as an association with breast cancer, mental illness, and infertility. These erroneous understandings were also more common among survey participants who had obtained an abortion, perhaps due to erroneous information received and/or medically inaccurate state mandated counseling during the clinical consultation.^9^

In other areas of healthcare, patients report delaying, or avoiding care due to concerns for perceived legal consequences. For example, fear of legal consequences is associated with avoidance of prenatal care among pregnant people who use illicit substances and fear of deportation induces undocumented immigrants to seek care outside of traditional healthcare facilities.^10^, ^11^, ^12^, ^13^, ^14^ We hypothesized that believing abortion is illegal would be associated with presentation for abortion care in the second trimester (≥14 weeks and 0 days) compared to the first trimester (<14 weeks and 0 days).

## METHODS

### Study setting and participant recruitment

This analysis comes from a larger survey‐based study conducted between April 2020 and April 2021, which aimed to capture the experiences of people seeking abortion in three neighboring US states that had similarly restrictive abortion policies at the time: Ohio, West Virginia, and Kentucky. We choose these three states due to their geographic proximity to one another and the imminent threat to abortion access in all three at the time of the study. We included in our analysis only those clinics that provided both first and second trimester care. The included clinics represent five of eight clinics in Ohio at the time of the survey, including all high‐volume clinics, and all abortion clinics in West Virginia (one) and one of two clinics in Kentucky. We excluded the second clinic in Kentucky as it does not provide second trimester abortion. At the time of the survey, abortion was legal, though heavily restricted, in all three states. For example, all three states had waiting period requirements between abortion counseling and procedure, gestational bans on abortion, and limitations on public funding of abortion.^15^

We based our recruitment goals for Ohio clinics on each clinic's patient volume in 2018. For Ohio clinics with lower patient volumes in 2018, we aimed to recruit at least 50 patients. For Ohio clinics with larger patient volumes in 2018, we aimed to recruit at least 150 patients. We chose these numbers in order to simplify messaging to clinics but did not cut off recruitment once they had met their recruitment goals. We aimed to recruit at least 150 patients from all clinics in West Virginia and Kentucky.^16^ We began recruitment in Ohio and then expanded to the neighboring states in the region.

Clinic staff distributed flyers describing the study to abortion seekers. Participants could be recruited at any visit, including during the mandatory counseling visit (as required by state law) or the separate abortion visit. Flyers advised prospective participants to complete the survey within 24 h of their appointment and each contained a unique link to a secure, online REDCap survey.^17^, ^18^ Eligible respondents had to be at least 18 years old, be seeking abortion care, be able to read English, and provide electronic consent. Participants received a $30 gift card. The Institutional Review Board (IRB) at the University of Cincinnati (UC) approved the project; The Ohio State University IRB ceded ethical review to the UC IRB through a reliance agreement.

### Data collection and key measures

The larger 80‐item survey collected participants' demographic, social, and behavioral information as well as their clinical experiences. This study focused on a subset of questions related to beliefs about abortion. To assess understanding of abortion legality among participants we asked, “When you were first deciding whether to have an abortion for this pregnancy, did you think abortion was legal or illegal in [the state that you live in]?” Answer choices included: “I thought it was legal,” “I thought it was illegal,” “I thought some abortions were legal, and others were illegal,” and “I did not know.” Participants self‐reported their gestational age and indicated whether dating was confirmed by ultrasound.

### Data analysis

Our primary research question focused on the relationship between participants' beliefs about abortion legality (exposure of interest) and timing of abortion care (outcome of interest). We coded legality as a binary variable by combining responses of “I thought it was legal” and “I thought some abortions were legal, and others were illegal” into a single “legal” category and combining responses of “I thought it was illegal” and “I did not know” into a single “illegal/unsure” category. We also coded timing of abortion care dichotomously: first trimester (<14 weeks and 0 days) or second trimester (≥14 weeks and 0 days). We analyzed data using Stata (Version 16, Stata Corp, TX). First, we examined associations between respondents' demographic/clinical factors (age, race, ethnicity, education, annual income, and prior abortion history) and their beliefs about abortion legality, using Pearson chi‐squared tests for categorical data and Mann–Whitney U tests or Kruskal‐Wallis tests for continuous data. Next, we specified unconditional logistic regression models to generate unadjusted and adjusted associations between believing abortion is illegal/unsure and receiving abortion care in the second trimester. In the adjusted model, we included variables previously associated with abortion timing, including age, race, ethnicity, birthplace, state of residence, education, income, and previous abortion history.^19^, ^20^, ^21^, ^22^, ^23^ In preliminary analyses, we assessed clustering by comparing models that included clinic as a fixed versus random effect. Because we did not observe meaningful clustering, we dropped clinic as a term from the final models.

To examine the robustness of the results of our primary analysis, we also conducted the following sensitivity analyses: (1) We repeated the primary analysis using our binary dependent variable (1st vs. 2nd trimester) but analyzed each of the four legality response options as separate categories, without combining into the two‐level legality variable using binary logistic regression; (2) We repeated the primary analysis using linear regression models to examine associations between legality beliefs and a continuous measure of gestational duration at termination (days gestation), rather than a dichotomous variable (first or second trimester); (3) We repeated the primary analysis and excluded the clinic that only provided first trimester abortion; and (4) We repeated the primary analysis using 10 weeks and 0 days as our gestational cutoff because after 10 weeks only instrumentation abortion is available in the states surveyed, and some patients may have preferred medication abortion care.^24^

## RESULTS

### Recruitment

**FIGURE 1:**
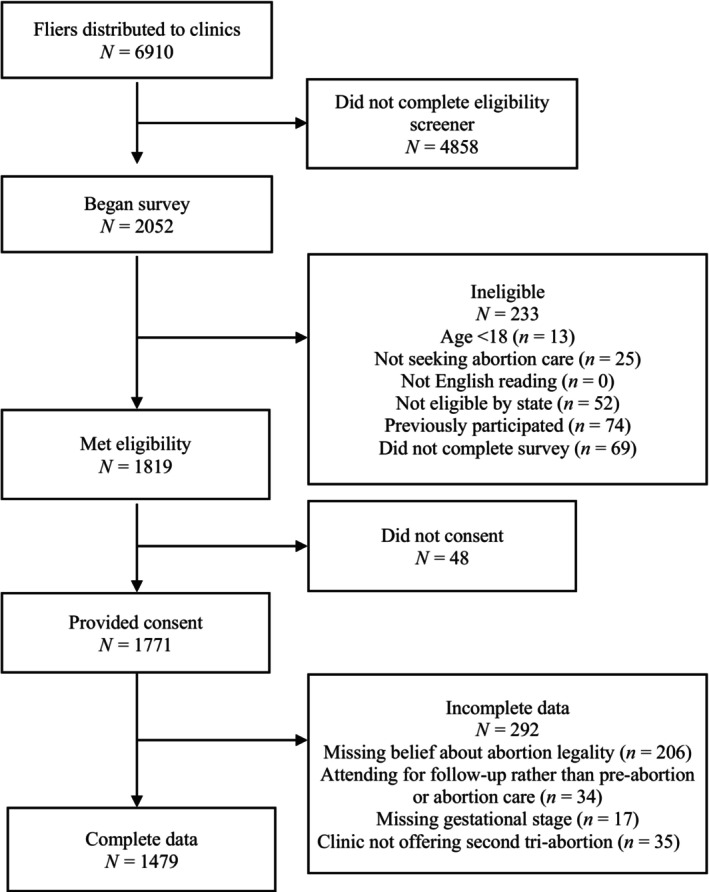
Study recruitment and inclusion among participants seeking abortion in Ohio, West Virginia and Kentucky.

Although we know the number of flyers issued to each clinic, we do not know how many patients received flyers nor the number of undistributed flyers at the end of the study. Thus, we calculated an approximate and conservative response rate: 6910 flyers were given to clinics for distribution to patients, 2052 patients completed the eligibility screener, and 1771 met our eligibility criteria and consented to participate, for a response rate of 26%. Of this sample, 1479 patients (84%) provided complete responses to the abortion legality and gestational duration questions and comprise the final analytic sample (Figure 1). Of these, 1287 (87%) reported having undergone an ultrasound evaluation.

### Sample characteristics

The median age of participants was 26 years (interquartile range [IQR]: 23–31 years). Most participants identified as Black (40%) or white (58%) and 5% reported Hispanic ethnicity. Most (94%) were born in the US and resided in Ohio (80%). Half of participants had completed some college or an associate degree (48%) while 29% reported completing high school or equivalent. Two‐thirds had at least one prior abortion (67%). Most participants (91%) presented for care in the first trimester of pregnancy (Table 1).

**TABLE 1:** Characteristics of participants seeking abortion in Ohio, West Virginia and Kentucky (*N* = 1479) overall and by abortion legality belief.

	Total	Legal/sometimes legal	Illegal/did not know	
	Median (IQR)	Median (IQR)	Median (IQR)	*p*‐value
Age	26 (23–31)	27 (23–31)	25 (22–30)	<0.01
Gestation (in days)	52 (44–67)	52 (44–67)	53 (44–67)	0.76

	Total		Legal/sometimes legal		Illegal/did not know		*p*‐value
	(*n* = 1479)		(*n* = 1153)		(*n* = 326)		
	*N*	(%)	*N*	(%)	*N*	(%)	
Race^a^							
Asian	34 (2)		29 (3)		5 (2)		0.30
Black	588 (40)		455 (39)		133 (41)		0.66
White	865 (58)		693 (60)		172 (53)		0.02
Other	72 (5)		52 (5)		20 (6)		0.23
Missing	39 (3)		20 (2)		19 (6)		<0.01
Ethnicity							<0.01
Hispanic/Latinx	80 (5)		53 (5)		27 (8)		
Non‐Hispanic/Latinx	1384 (94)		1091 (95)		293 (90)		
Missing	15 (1)		9 (1)		6 (2)		
Birthplace							0.09
US born	1392 (94)		1093 (95)		299 (92)		
Not US born	64 (4)		43 (4)		21 (6)		
Missing	23 (2)		17 (1)		6 (2)		
State of residence							0.09
Ohio	1180 (80)		907 (79)		273 (84)		
West Virginia	141 (10)		119 (10)		22 (7)		
Kentucky	158 (11)		127 (11)		31 (10)		
Annual income							<0.01
$0–9999	307 (21)		226 (20)		81 (25)		
$10 k–29,999	577 (39)		453 (39)		124 (38)		
$30 k–49,999	284 (19)		225 (20)		59 (18)		
$50 k+	203 (14)		173 (15)		30 (9)		
Missing	108 (7)		76 (7)		32 (10)		
Education							<0.01
0–11th grade	70 (5)		45 (4)		25 (8)		
High school graduate/GED	435 (29)		313 (27)		122 (37)		
Some college/associate degree	713 (48)		569 (49)		144 (44)		
College graduate or higher	257 (17)		222 (19)		35 (11)		
Missing	4 (0.3)		4 (0.4)		0 (0)		
Abortion history							0.08
No prior abortion	480 (32)		359 (31)		121 (37)		
Prior abortion	997 (67)		793 (69)		204 (63)		
Missing	2 (0.1)		1 (0.1)		1 (0.3)		
Trimester of abortion							0.45
First trimester	1350 (91)		1049 (91)		301 (92)		
Second trimester	129 (9)		104 (9)		25 (8)		

*Note*: *p*‐value determined using Pearson chi‐squared tests for categorical data and Mann–Whitney U tests or Kruskal‐Wallis tests for continuous data.

Abbreviations: GED, general educational development test; IQR, interquartile range; US, United States.

^a^ Participants could select more than one category so responses may sum to >100%.

### Abortion legality belief

Before seeking abortion care, most respondents believed abortion was always (57%) or sometimes (21%) legal, while a minority believed abortion was illegal (12%) or did not know whether abortion was legal (10%). Compared to participants who believed abortion was legal or sometimes legal, those who believed abortion was illegal or were uncertain about legality were younger (median age 25, IQR 22–30 vs. median age 27, IQR 23–31; *p* < 0.01), more likely to report Hispanic ethnicity (8% vs. 5% *p* = 0.02), lower income (25% vs. 20% of $0–9999 income category; *p* < 0.01), and lower educational attainment (37% vs. 27% of High school graduate/General Educational Development Test education category; *p* < 0.01) (Table 1). Participants who believed abortion was legal or sometimes legal were more likely to report white race compared to Asian, Black, or another race (60% vs. 53% white, 3% vs. 2% Asian, 39% vs. 41% Black, 5% vs. 6% other; *p* = 0.03) (Table 1).

### Abortion legality belief and gestational age at presentation for abortion services

We found no significant associations between belief that abortion was illegal, or uncertainty of legality, and timing of abortion care (unadjusted odds ratio for second trimester abortion [uOR]: 0.78; 95% confidence interval [CI]: 0.50–1.20) (Table 2). After adjusting for age, race, ethnicity, nationality, state of residence, education, income, and previous abortion history, this association remained largely unchanged (adjusted odds ratio [aOR]: 0.83; 95% CI: 0.51–1.33) (Table 2).

**TABLE 2:** Unadjusted and adjusted associations between abortion legality beliefs and receiving abortion care in the second trimester among participants receiving abortion care in Ohio, West Virginia and Kentucky (*N* = 1479).

	OR	(95% CI)
Unadjusted		
Legal versus sometimes legal	0.79	(0.51–1.21)
Illegal versus sometimes legal	0.58	(0.29–1.16)
Didn't know versus sometimes legal	0.82	(0.42–1.61)
Adjusted^a^		
Legal versus sometimes legal	0.84	(0.52–1.36)
Illegal versus sometimes legal	0.57	(0.28–1.16)
Didn't know versus sometimes legal	0.71	(0.33–1.53)

Abbreviations: CI, confidence interval; OR, odds ratio.

^a^ Adjusted for state of residence, age, race, ethnicity, income, education, abortion history, and nationality.

In a sensitivity analysis modeling each legality response option separately, we compared each category to the referent group of believing abortion was sometimes legal. We found no association between believing abortion was legal (uOR: 0.83, 95% CI: 0.51–1.33), believing abortion was illegal (uOR: 0.57, 95% CI: 0.28–1.18) and being unsure of abortion legality (uOR: 0.72, 95% CI: 0.34–1.57) and having a second trimester abortion compared to believing abortion was sometimes legal. This lack of association persisted after adjustment for state of residence, age, race, ethnicity, income, education, abortion history, and nationality.

The sensitivity analysis modeling the association between beliefs about abortion legality and gestation at abortion as a continuous variable also yielded similar results. People who believed abortion was illegal or were unsure received abortion care at approximately the same gestation as those who believed abortion was legal or sometimes legal (unadjusted difference: 0.17 days, 95% CI: −2.7 days to 3.1 days; adjusted difference: −0.95 days, 95% CI: −4.0 to 2.2 days). The sensitivity analysis modeling that used 10 weeks 0 days as a gestational cutoff yielded a similar lack of association (data not shown).

## DISCUSSION

In our study we did not find an association between belief that abortion is illegal or uncertainty and abortion care later in pregnancy, which we confirmed through our sensitivity analyses. The lack of association with abortion timing, while surprising, may indicate that abortion legality concerns impact timing of presentation in unexpected ways, such as by inducing patients with concerns over abortion legality to seek care earlier rather than later due to fear of being unable to access care. In addition, in the year prior to data collection, Ohio had passed a law that banned all abortion following embryonic cardiac‐activity, or roughly 6‐weeks of gestation. After passing, this law was immediately blocked by a federal judge. Such a shifting legal landscape likely augmented uncertainty and may have affected our results in complex ways.

Abortion is a safe procedure, but receiving care at a later gestational age increases medical complications and financial costs.^25^, ^26^, ^27^, ^28^ Abortion most commonly occurs in the first trimester.^19^ Although abortion seekers generally prefer to have abortions earlier in pregnancy, scheduling difficulties, familial demands, financial burdens, and laws requiring waiting periods may delay abortion care.^20^ Adolescents, Black and Hispanic women experiencing structural racism, and abortion seekers living on lower incomes, with lower educational attainment, and/or living farther from an abortion‐providing facility are also more likely to abortion care in the second trimester.^20^, ^21^, ^22^, ^23^

Consistent with a population‐based survey of adult, reproductive age women in Ohio, a cross‐sectional nationally representative sample of reproductive age adult women, and a survey of Texas women, we found that before seeking care, a significant minority—more than 20%—of patients who were receiving abortion care nevertheless believed that care to be illegal or were unsure of its legality.^6^, ^29^, ^30^ Unlike prior studies that surveyed the general population, we surveyed abortion seekers. Despite believing that abortion was illegal or being unsure, our participants still sought abortion care.

In our sample, compared to those who thought abortion was legal or sometimes legal, patients who believed abortion to be illegal or were unsure of its legality were more likely to be younger, report Hispanic ethnicity, be living on a lower income, and have lower educational attainment. Those who believed abortion was legal or sometimes legal were more likely to report white race compared to Black, Asian, or another race. These demographic associations are similar to those of a representative sample of Ohioans which found associations between younger age, low socioeconomic status, and reporting Black race and believing abortion to be illegal.^6^

### Limitations

Although we enrolled a large and diverse sample from abortion clinics in three abortion‐restrictive states, we only surveyed patients presenting for care and did not include people who did not present for abortion care, including those who never presented for care because they believed abortion was illegal. We also did not survey those people who obtained an abortion outside of the formal health care system, those who continued an unwanted pregnancy, nor those who traveled out of the three states in our study to obtain abortion care—potentially because of their beliefs regarding abortion illegality or later gestational stage. Additionally, patients with time and resources to complete our survey may disproportionately participate in survey research. Similarly, patients who understood that abortion is legal may be more willing to participate in a survey about abortion legality compared to those patients with concerns about abortion legality. Based on these selection biases, we may not have been able to fully capture data from those whose concerns about illegality led them to delay abortion care.

Our participants all resided in Ohio, West Virginia and Kentucky, states with highly restrictive abortion laws even before the Dobbs decision.^31^ Statewide debate on abortion may have contributed to some participants' beliefs about abortion legality, even among those presenting for care. Findings from our study may not be generalizable to states with fewer abortion restrictions.

### Priorities for future research

To our knowledge, this is the first survey to assess the relationship between abortion legality beliefs and timing of abortion among abortion seekers. Future research should target pregnant women, transgender men, and gender nonbinary individuals who do not present for abortion care due to beliefs that abortion is illegal, to generate a more comprehensive estimate of the prevalence of abortion legality beliefs and any association with delays in care presentation. Our survey did not ask participants when they learned that abortion was legal and individuals seeking abortion care may also use social networks or online information to understand better abortion legality when seeking abortion care. Future work should explore how individuals obtain information on abortion legality.

Additional research could also explore how educational interventions through public health campaigns regarding abortion legality could aid abortion seekers.^16^ Legality, and perceived legality, is associated with abortion stigma.^31^ Abortion stigma may influence an abortion seeker's decision to prioritize secrecy rather than safe care and result in adverse outcomes.^32^, ^33^, ^34^ Amid mounting abortion restrictions future studies should consider associations between stigma, understanding of legality, and timing of abortion care. Finally, perceptions about abortion legality may have changed since Dobbs. Future studies could explore how confusion regarding abortion legality have shifted since Dobbs.

### Implications for practice and/or policy

That patients in our study who believed that abortion was illegal still presented for and obtained safe, legal care demonstrates the determination of abortion seekers to exercise reproductive autonomy. Increasing state restrictions post‐Dobbs could exacerbate confusion, increase stigma, and impede access to safe abortion care, particularly in states where at least some abortions remain legal.^32^

Additionally, belief that abortion is illegal or uncertainty about legality could contribute to an increased number of women, transgender men, and gender nonbinary individuals accessing abortion outside of the formal medical system. Patients can self‐manage their abortions safely by self‐sourcing the same drugs used for medication abortion in the formal healthcare system or induce an abortion unsafely through use of toxic substances, self‐injury, or uterine trauma.^34^, ^35^, ^36^ Safe self‐managed abortion has been and will continue to be essential for abortion seekers who struggle to access abortion care through the formal healthcare system, whether because of legal restrictions, abortion stigma, or a desire for more private abortion care (though self‐managed abortion could bring legal risk).^36^, ^37^, ^38^ Those who believe abortion is illegal or are uncertain may increasingly rely options outside of the formal health system especially in states where abortion is heavily restricted.^37^, ^38^, ^39^

## CONCLUSION

Although abortion was legal in Ohio, West Virginia and Kentucky at the time of our survey, a substantial minority of our survey participants believed that abortion was illegal or were uncertain about legality prior to accessing abortion care. With increasing restrictions and near complete abortion bans in some states in the wake of the Dobbs decision, confusion over abortion's legal status will only grow and may discourage patients from accessing safe, legal services. Efforts to educate the public on local abortion restrictions and advocate for improved access to abortion care are of critical importance.

## FUNDING INFORMATION

This study was supported by an Anonymous Foundation. The funder had no involvement in study design, data collection, analysis or data interpretation, nor in manuscript writing or the decision to submit the manuscript for publication.

## CONFLICT OF INTEREST STATEMENT

The authors report no conflicts of interest.
